# Immunophenotyping patients with sepsis and underlying haematological malignancy reveals defects in monocyte and lymphocyte function

**DOI:** 10.1186/s40635-023-00578-4

**Published:** 2024-01-11

**Authors:** Timothy Arthur Chandos Snow, Aimee Serisier, David Brealey, Mervyn Singer, Nishkantha Arulkumaran, University College London Hospitals Critical Care Research Team, Naveed Saleem, Antonio Cesar, Alessia V. Waller, Francis Ryckaert, Deborah Smyth, Georgia Bercades, Ingrid Hass, Alexandra Zapata Martinez, Laura Gallagher, Gladys Martir

**Affiliations:** Bloomsbury Institute of Intensive Care Medicine, University College London, 1.1 Cruciform Building, Gower Street, London, WC1E 6DH UK

**Keywords:** Sepsis, Haematology, Intensive care unit, Monocyte, Lymphocyte

To the Editor,

Sepsis is a common reason for intensive care unit (ICU) admission of patients with haematological malignancy [1]. The main focus is placed on neutropenia, with little attention paid to other white cell lineage such as monocytes and lymphocytes. Immune dysfunction in these cells is well-described in non-cancer septic patients and associated with an increased mortality risk [2–4]. Features typically associated include impaired monocyte antigen presentation and co-stimulation (HLA-DR, CD80, CD86), increased immune checkpoint inhibition (lymphocyte PD-1 and monocyte PD-L1), impaired lymphocyte proliferation/ maturation (IL-7 receptor), activation (CD28 and CTLA-4), and viability [2–4]. The primary objective of this feasibility study was to ascertain whether these cells are similarly affected in haematology patients with sepsis.

We conducted a prospective observational study in patients with or without haematological malignancy admitted to the ICU with sepsis. Peripheral blood mononuclear cells (PBMC) were isolated and assessed by multi-parameter flow cytometry, and serum immune analytes by ELISA (Additional file 1: Methods). A focused analysis was performed of cell surface markers associated with sepsis-induced immunosuppression [2–4].

We included 11 haematology ICU patients, 33 non-haematology ICU patients (and 17 healthy volunteers as a reference). Patient demographics are detailed in Additional file 1: Table S2. Compared to non-haematology patients, haematology patients were of similar age and had a similar SOFA score. However, compared to non-haematology patients, haematology patients had lower neutrophils (*p* < 0.0001), lymphocytes (*p* = 0.03), and monocytes (*p* = 0.005). Hospital mortality was similar between both groups (27% non-haematology vs. 36% haematology) (Fig. 1, Additional file 1: Fig. S1).

**Fig. 1:**
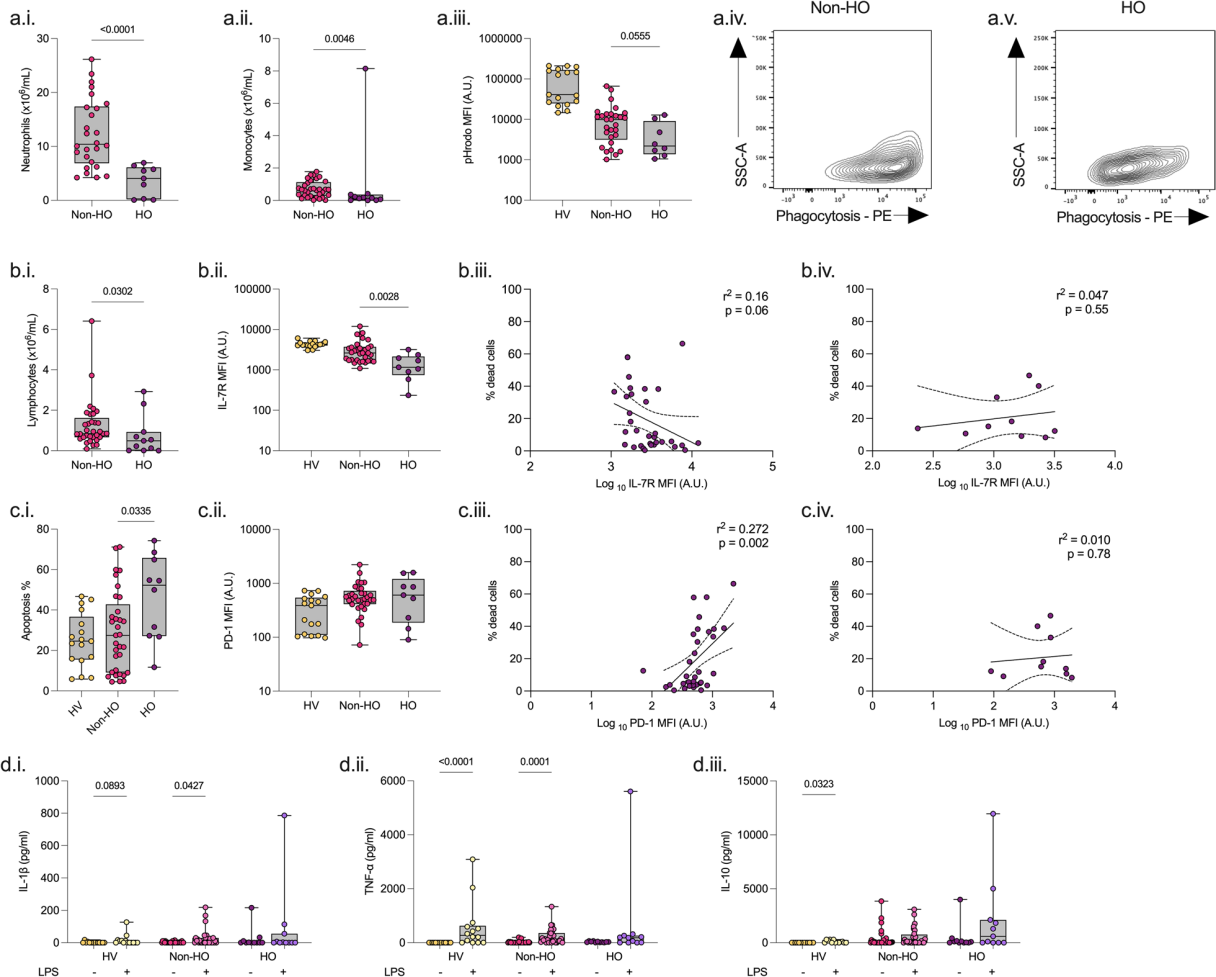
Differences in clinical variables, monocyte and lymphocyte function between non-haematology and haematology patients. Comparison of patients admitted to the Intensive Care Unit with a non-haematology (Non-HO, *n* = 33), or haematology (HO, *n* = 11) diagnosis. Healthy volunteers (*n* = 17) are included as a reference. Innate immune response (**a.**) including neutrophil count (**i.**), monocyte count (**ii.**), and monocyte phagocytosis as measured by pHRodo (**iii.**), with example contour plot of non-HO (**iv.**) and HO (*v.*). Adaptive immune response (**b.**–**c.**) including lymphocyte count (**b.i.**) CD4 lymphocyte IL-7 receptor (IL-7R) expression (**b.ii.**) and correlation plot of IL-7R with percentage cell death of non-HO (**b.iii.**) and HO (**b.iv.**), apoptosis (c.i.), and programmed cell death receptor-1 (PD-1) expression (**c.ii.**) correlation plot of PD-1 with percentage cell death of non-HO (**c.iii.**) and HO (**c.iv.**) patients. LPS-induced cytokine release (**d.**) including IL-1β (**i.**), TNF-α (**ii.**) and IL-10 (**iii.**). Data compared using Mann Whitney test. Only *p* < 0.1 shown

There was a trend towards decreased monocyte phagocytosis (*p* = 0.055) among haematology patients. Viability in lymphocyte CD4 and CD8 cell populations and CD4 IL-7R levels were lower among haematology patients (Fig. 1, Additional file 1: Figs. S2, S3). A positive correlation was seen between PD-1 expression and cell death in CD4 lymphocytes in non-haematology patients but not haematology patients (Fig. 1).

Serum TNF-α was higher among haematology patients, although monocyte intracellular TNF-α levels were similar. Following ex vivo whole blood stimulation with LPS, serum IL-1β (*p* = 0.043) and TNF-α (*p* = 0.001) increased significantly in non-haematology patients, but not in haematology patients. (Fig. 1).

We present novel data demonstrating immune dysfunction in monocytes and lymphocytes taken from haematology patients with sepsis; over and above that seen in non haematology patients. This included impaired monocyte phagocytosis, and impaired release of TNF-α and IL-1β (canonical cytokines associated with monocyte function) on whole blood stimulation with LPS. Intriguingly, monocyte HLA-DR, a robust functional marker of immunoparesis in critically ill patients [4], was not different in haematology patients.

Mechanisms of lymphocyte death are likely to differ between haematology and non-haematology patient cohorts. The association between CD4 lymphocyte PD-1 expression and cell death is also described in patients with sepsis [4]. We found a positive correlation between PD-1 expression in CD4 lymphocytes in non-haematology patients but not in haematology patients.

Existing therapies to improve clinical outcomes in the critically ill haematology patient with sepsis are limited. Further research is required to gain a better understanding of the immune phenotype in this population, providing a rational for individualized sepsis treatment.

### Supplementary Information

**Additional file 1: Table S1.** Flow cytometry fluorochromes used. **Table S2.** Baseline demographics. **Figure S1.** Differences in laboratory-measured variables between non-haematology and haematology patients. **Figure S2.** Differences in classical monocyte and CD4^+^ and CD8^+^ lymphocyte function between non-haematology and haematology patients. **Figure S3.** Differences in classical monocyte and CD4^+^ and CD8^+^ lymphocyte function between non-haematology and haematology patients.

## Data Availability

Available upon reasonable request and at discretion of investigators’ institution.
