# Supplementary material for: Immunophenotyping patients with sepsis and underlying haematological malignancy reveals defects in monocyte and lymphocyte function
Source: Intensive Care Med Exp. 2024 Jan 11;12:3. doi: 10.1186/s40635-023-00578-4 (PMC10784430; doi:10.1186/s40635-023-00578-4)
Supplement: Supplementary file 1 — Additional file 1: Table S1. Flow cytometry fluorochromes used. Table S2. Baseline demographics. Figure S1. Differences in laboratory-measured variables between non-haematology and haematology patients. Figure S2. Differences in classical monocyte and CD4+ and CD8+ lymphocyte function between non-haematology and haematology patients. Figure S3. Differences in classical monocyte and CD4+ and CD8+ lymphocyte function between non-haematology and haematology patients. [file 40635_2023_578_MOESM1_ESM.docx]

# Additional Material

### Additional Methods

We conducted a prospective observational study of patients aged ≥18 years admitted to the Intensive Care Unit (ICU) with sepsis at University College London (UCL) Hospitals between 1^st^ August 2021 and 31^st^ July 2022. Patient demographics, clinical data (physiology, diagnoses), laboratory data, and clinical outcomes were recorded from electronic healthcare records. Diagnosis of sepsis was made by the treating clinicians based on the Sepsis-3 criteria.

Blood samples were taken from these patients within the first week of their ICU admission. As this was a feasibility study, sample size was not calculated.

Following patient recruitment, venesection was performed at time of taking blood culture and 8mls of blood drawn into a Cell preparation tube (CPT^TM^) vacutainer and a further 5mL drawn into a serum and EDTA tube (all Beckton Dickinson (BD), Wokingham, UK). Samples were processed within 1hour of venesection. CPT^TM^ vacutainers were centrifuged at 1500 relative centrifugal force (rcf) for 15minutes at room temperature and the PBMC layer extracted, washed twice in 2mls of phosphate buffered saline (PBS) before being resuspended in freezing media (bovine serum albumin with 10% dimethyl sulfoxide (DMSO)) and frozen to -80^o^C in a Mr Frosty^TM^ and transferred to liquid nitrogen within 48hours for long term storage.

To measure released cytokines, 500ul of EDTA blood was stimulated for 1hour with 100ng/ml LPS. Serum tubes and stimulated samples were then centrifuged for 15mins at 1500rcf and the serum aspirated and stored frozen for subsequent analysis.

Samples were analysed in batches. Frozen PBMCs were defrosted by resuspension in RPMI Glutamax medium (Gibco, Thermo Fisher (TF), Cambridge, UK) with 10% foetal bovine serum (FBS) (TF), washed once in media, counted (Countess 3 Automated cell counter, TF), and diluted to a concentration of 1x10^6^/ml.

To assess monocyte cell surface antigens, PBMCs were resuspended in PBS and incubated with relevant antibodies (CD14, CD16, HLA-DR, CD80, CD86, and CD274 (PD-L1), and viability stain (Aqua UV Live/Dead). After 30mins PBMCs were fixed and permeabilised using the CytoFix/Perm kit (BD) after which they were resuspended and incubated in Cytoperm/Wash (BD) with intracellular cytokines. Monocyte phagocytosis was assessed by incubation of PBMCs with pHRodo opsonised bioparticles (TF) for 1hour prior to labelling with cell surface markers (CD14, CD16, HLA-DR, and Blue UV Live/Dead). Cytokine release following *ex vivo* LPS stimulation of whole blood were also measured.

To assess lymphocyte viability and cell surface antigens, PBMCs were resuspended in annexin buffer (BD) and relevant antibodies (CD3, CD4, CD8, CD19, CD25 (IL-2RA), CD28, CD127 (IL-7RA), CD152 (CTLA-4), CD274 (PD-L1), and CD279 (PD-1)) with viability stain (Aqua UV Live/Dead and Annexin V). Details of products and concentrations used are detailed in Table S1.

Cells were acquired on an LSR II flow cytometer (BD) running BD FACSDiva version 9. Calibrations beads (BD) were run prior to each experiment and compensation controls were applied to all samples prior to analysis. Single-stained unstimulated healthy donor cells were used as compensation controls for cell surface markers. Healthy donor cells for the were heat-treated at 60^o^C for 10 minutes as a positive control for cell death. Compensation beads (BD) were used as positive controls for intracellular cytokines. FMO (fluorescence minus one) samples for all fluorophores were used to identify cell populations. Cell populations of interest were identified using the following Boolean gating strategy: lymphocytes or PBMCs, singlets, live cells, and cell surface markers and stopping gate set at 10,000 events for either CD14^hi^CD16^lo^ monocytes or CD4^+^ lymphocytes. Flow cytometry data were analysed using FlowJo (version 10.7.1, BD). Samples with cell counts fewer than 50 in the population of interest were excluded.

We measured levels of IL-1β, IL-6 IL-10, TNF-α, PD-1, and PD-L1 in patient serum using Duoset ELISA kits (R&D Systems, Minneapolis, MN) as per manufacturer instructions. Samples were diluted 1:2 in reagent dilutant. Optical densities were acquired on a SPECTROstar Nano microplate reader (BMG Labtech, Aylesbury, UK).

Clinical and demographic data are presented either as median (inter-quartile range) or number (percentage). Flow cytometry data are presented as either median fluorescence intensity (MFI; arbitrary units) or percentage positive cells with interquartile ranges. Comparison between patients with or without haematological malignancy was analysed using Mann Whitney or chi-squared test for continuous and categorical data respectively.

| **Table S1: Flow cytometry fluorochromes used** | | | |
| --- | --- | --- | --- |
| Antibody | **Fluorochrome** | **Catalogue no** | **[Final]** |
| CD14 | BV785 | BL 301840 | 1:250 |
| CD16 | BUV395 | BD 563785 | 1:250 |
| HLA-DR | APC-Cy7 | BL307618 | 1:250 |
| LD Aqua | Aqua UV | TF L34957 | 1:1000 |
| CCR2 | BV711 | BL 357232 | 1:250 |
| CXCR4 | BV421 | BL 306518 | 1:250 |
| CD80 | PE | BL 305208 | 1:250 |
| CD86 | PE-Dazzle | BL 374218 | 1:250 |
| CD274 | APC | BD 563741 | 1:250 |
| IL-1β | FITC | TF 11-7018-42 | 1:100 |
| IL-6 | PerCP-Cy5.5 | BL 501118 | 1:100 |
| IL-10 | PE-CY7 | BL 501420 | 1:100 |
| TNF-α | BUV737 | TF 367-7349-42 | 1:100 |
| pHRodo Red | PE | TF P35361 | 100ug/ml |
| CD3 | BUV395 | BD 564001 | 1:250 |
| CD4 | BV785 | BL 317442 | 1:250 |
| CD8 | BV711 | BD 563677 | 1:250 |
| CD19 | APC-Cy7 | BD 557791 | 1:250 |
| LD Blue | Blue UV | TF L34962 | 1:1000 |
| Annexin | AF 350 | TF A23202 | 1:250 |
| CD25 | BV421 | BD 562442 | 1:250 |
| CD28 | BUV737 | BD 748475 | 1:250 |
| CD127 | PE | BL 351304 | 1:250 |
| CD152 | PE-Dazzle | BL 349922 | 1:250 |
| CD274 | APC | BD 563741 | 1:250 |
| CD279 | PE-Cy7 | BD 561272 | 1:250 |
| Abbreviations: CD: Cluster of Differentiation; HLA-DR: Human leukocyte antigen – DR isotype; LD: Live/Dead; CCR2: C-C motif chemokine receptor 2; CXCR4: C-X-C motif chemokine receptor 4; IL: Interleukin; TNF: Tissue necrosis factor; IFN: Interferon; BL: Biolegend; BD: Beckton Dickinson; TF: Thermo Fisher | | | |

| **Table S2: Baseline demographics** | | | | | |
| --- | --- | --- | --- | --- | --- |
| Variable | | **Healthy volunteer (n=17)** | **Non- Haem-onc (n=33)** | **Haem-onc (n=11)** | **p-value** |
| Age (years) | | 37 (31-44) | 56 (44-73) | 57 (46-65) | 0.7434 |
| Sex (% male) | | 88% | 64% | 64% | 0.1680 |
| Ethnicity | | | | | |
|  | Asian | 35% | 18% | 9% | 0.4278 |
|  | Black | 0% | 15% | 18% |  |
|  | White | 59% | 64% | 64% |  |
|  | Other | 6% | 3% | 9% |  |
| SOFA Score | | - | 6 (4-9) | 6 (5-10) | 0.6943 |
| Blood glucose | | - | 7.5 (4.3-9.2) | 9.3 (6.8-10.0) | 0.3147 |
| Haem-oncology diagnosis | |  | | | |
|  | Acute myeloid leukaemia | - | - | 27% | - |
|  | B-cell lymphoma | - | - | 9% | - |
|  | T-cell lymphoma | - | - | 9% | - |
|  | Mantle-cell lymphoma | - | - | 18% | - |
|  | Follicular cell lymphoma | - | - | 9% | - |
|  | Myelodysplastic syndrome | - | - | 27% | - |
| Admission haem-oncology treatment(s) | | | | | |
|  | Chemotherapy | - | - | 73% | - |
|  | Immunotherapy | - | - | 45% | - |
|  | Stem cell transplant | - | - | 27% | - |
|  | Chimeric antigen receptor T-cell (CAR-T) therapy | - | - | 9% | - |
| Mortality (%) | | - | 27% | 36% | 0.7058 |
| Data compared using Mann Whitney U, or chi-squared test for continuous and categorical data respectively. | | | | | |


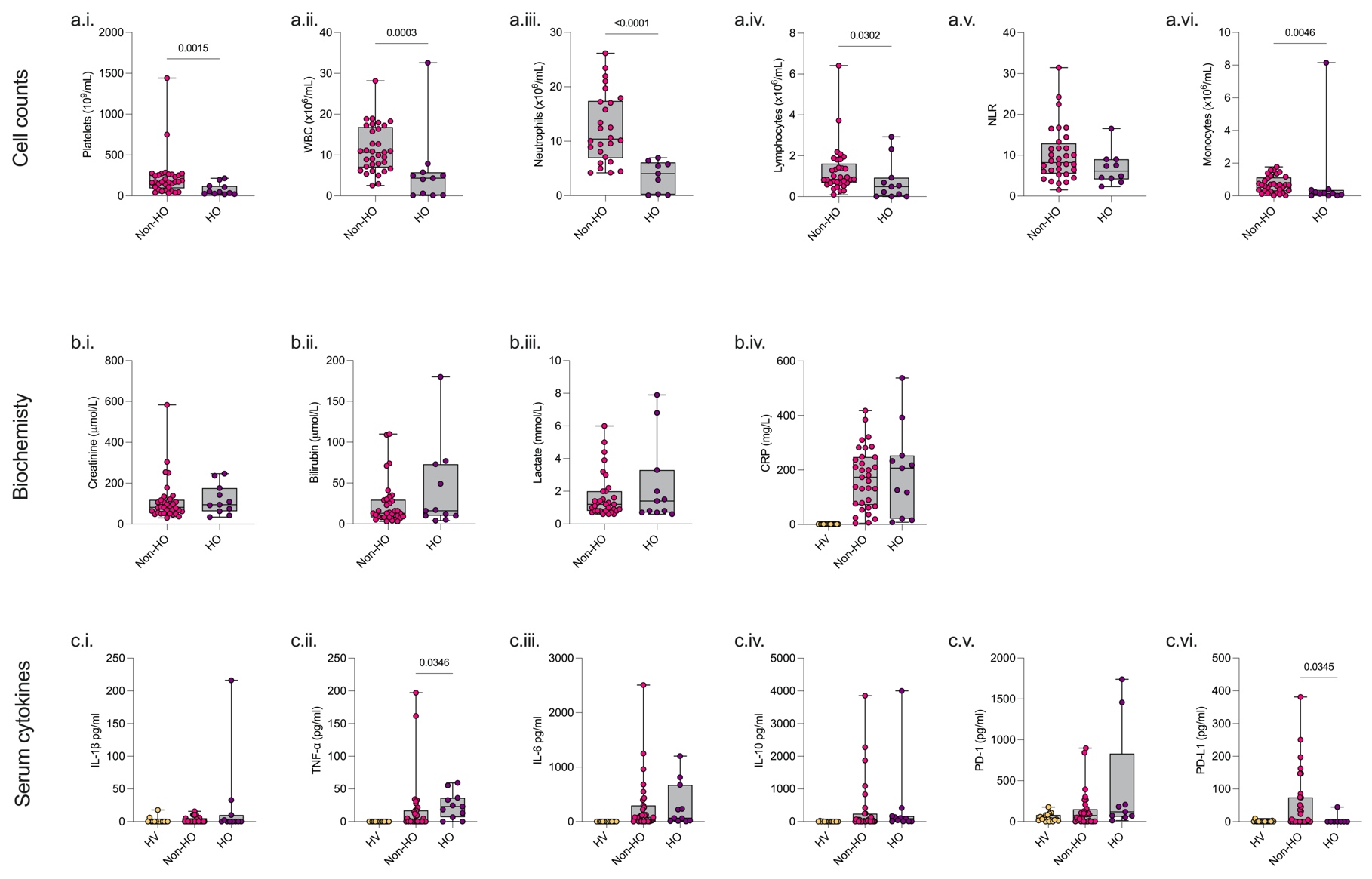


### Figure S1: Differences in laboratory-measured variables between non-haematology and haematology patients

Comparison of patients admitted to the Intensive Care Unit with a non-haematology (Non-HO, n=33), or haematology (HO, n=11) diagnosis. Healthy volunteers (n=17) are included as a reference for cytokines. Variables include routine laboratory measured cell counts (a. platelets (i.), white cells (ii.), neutrophils (iii.), and lymphocytes (iv.), neutrophil-lymphocyte ratio (NLR, v.), and monocytes (vi.)), routine laboratory measured biochemistry (b. creatinine (i.), bilirubin (ii.) lactate (iii), and C-reactive protein (CRP, iv.)) , and serum cytokines (c. interleukin-1β (IL-1β, i.), tissue necrosis factor-α (TNF-α, ii.), IL-6 (iii.), IL-10 (iv.), programmed cell death receptor-1 (PD-1, v.) and programmed cell death ligand-1 (PD-L1, vi.)). Data compared using Mann Whitney test. Only p<0.1 shown.


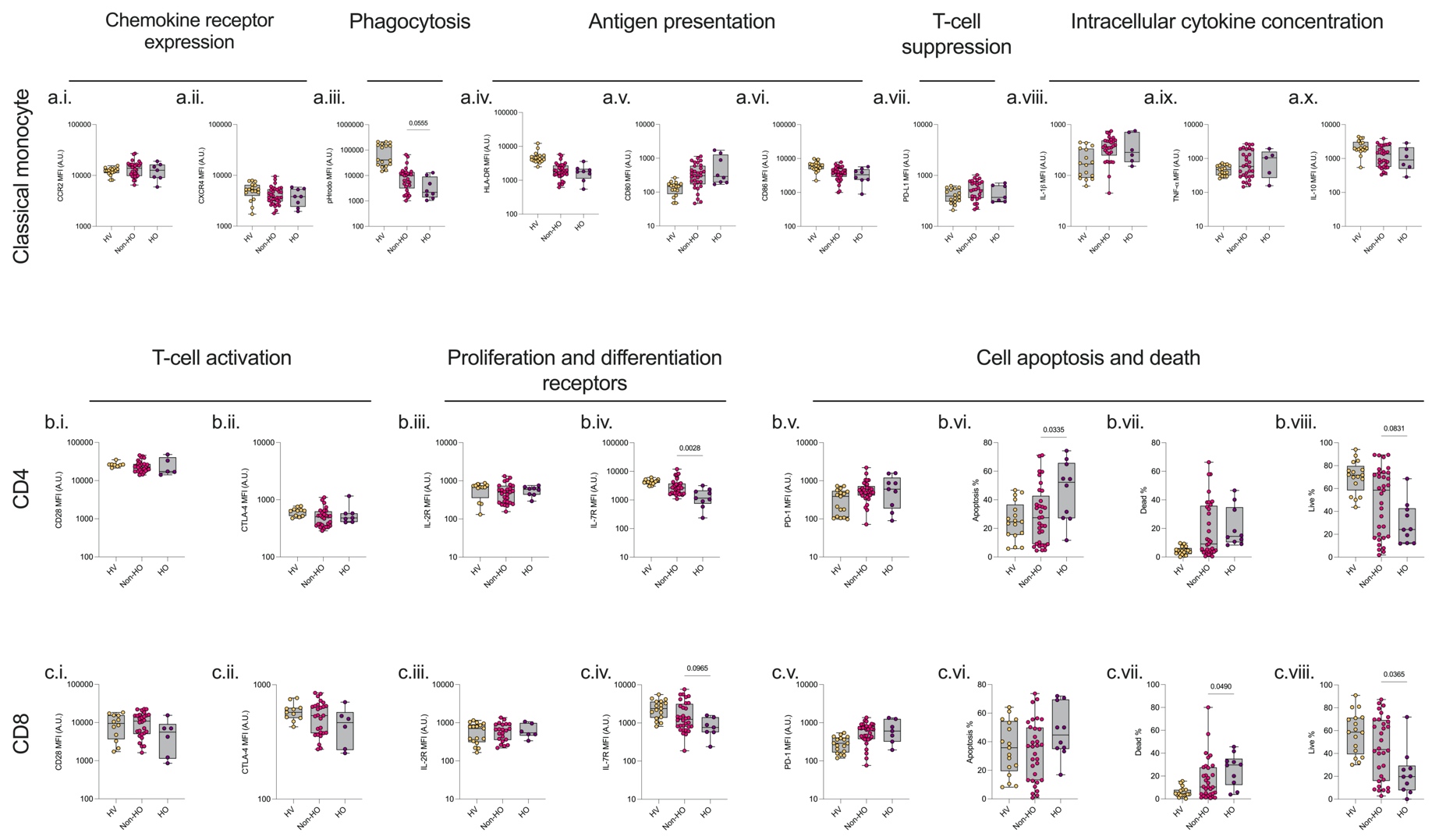


### Figure S2: Differences in classical monocyte and CD4^+^ and CD8^+^ lymphocyte function between non-haematology and haematology patients

Comparison of patients admitted to the Intensive Care Unit with a non-haematology diagnosis (Non-HO, n=33), or haematology (HO, n=11) diagnosis. Healthy volunteers (n=17) are included as a reference. Variables including classical monocyte chemokine receptor expression (C-C motif chemokine receptor-2 (CCR2, a.i.) and C-X-C motif chemokine receptor-4 (CXCR4, a.ii.)), phagocytosis (measured using pHRodo bioparticles, a.iii.) antigen presentation (Human leukocyte antigen – DR isotype (HLA-DR, a.iv.), CD80 (a.v.), and CD86 (a.vi.)), T-cell suppression (programmed cell death ligand-1 (PD-L1, a.vii)) and intracellular cytokine concentration (interleukin-1β (IL-1β, a.viii), tissue necrosis factor-α (TNF-α, a.xi), and IL-10 (a.xii.)). CD4^+^ (b.) and CD8^+^ (c.) lymphocyte activation (CD28 (i.) and cytotoxic T-lymphocyte associated protein-4 (CTLA-4, ii.)), differentiation and proliferation (IL-2 (iii.) and IL-7 (iv.) receptors), and cell death (programmed cell death receptor-1 (PD-1, v.), apoptosis (vi.), death (vii.) and live (viii.). Data presented as percentage positive cells (apoptosis, death and live) or median fluorescent intensity (MFI) and compared using Mann Whitney test. Only p<0.1 shown.


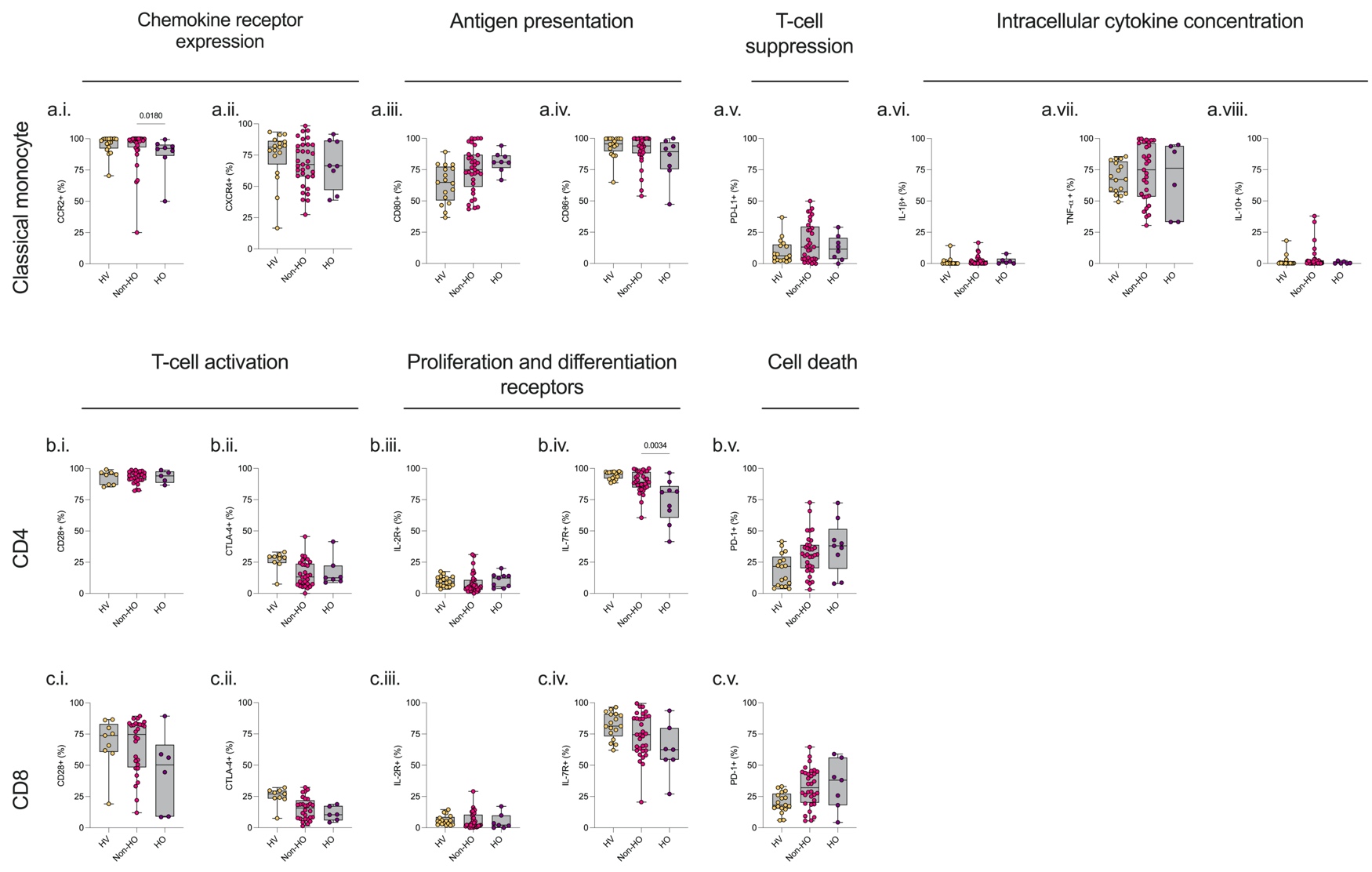


### Figure S3: Differences in classical monocyte and CD4^+^ and CD8^+^ lymphocyte function between non-haematology and haematology patients

Comparison of patients admitted to the Intensive Care Unit with a non-haematology diagnosis (Non-HO, n=33), or haematology (HO, n=11) diagnosis. Healthy volunteers (n=17) are included as a reference. Variables including classical monocyte chemokine receptor expression (C-C motif chemokine receptor-2 (CCR2, a.i.) and C-X-C motif chemokine receptor-4 (CXCR4, a.ii.)), antigen presentation (CD80 (a.iii.), and CD86 (a.iv.)), T-cell suppression (programmed cell death ligand-1 (PD-L1, a.v)) and intracellular cytokine concentration (interleukin-1β (IL-1β, a.vi), tissue necrosis factor-α (TNF-α, a.vii), and IL-10 (a.viii.)). CD4^+^ (b.) and CD8^+^ (c.) lymphocyte activation (CD28 (i.) and cytotoxic T-lymphocyte associated protein-4 (CTLA-4, ii.)), differentiation and proliferation (IL-2 (iii.) and IL-7 (iv.) receptors), and cell death (programmed cell death receptor-1 (PD-1, v.)). Data presented as percentage positive and compared using Mann Whitney test. Only p<0.1 shown.
